# Comparison of Radiography with Computed Tomography and Magnetic Resonance Imaging in the Measurement of Cervical Lordosis

**DOI:** 10.3390/medicina61091654

**Published:** 2025-09-11

**Authors:** Ismail Ertan Sevin, Selin Bozdag, Efecan Erisken, Hasan Kamil Sucu

**Affiliations:** Department of Neurosurgery, Ataturk Training and Research Hospital, Izmir Katip Çelebi University, İzmir 35620, Turkey; seviner67@yahoo.com (I.E.S.); selin.bzdg@gmail.com (S.B.); efecaneriskenn@gmail.com (E.E.)

**Keywords:** cervical lordosis, computed tomography, magnetic resonance imaging, standing lateral radiograph, Cobb angle, sagittal alignment

## Abstract

*Background and Objectives*: The assessment of cervical lordosis is essential for surgical planning and outcome prediction in patients with cervical spine pathology. This study aims to evaluate the accuracy of cervical lordosis measurements obtained on supine CT and MRI relative to standing lateral radiographs. *Materials and Methods*: In this retrospective review, 108 patients who underwent standing lateral radiographs, supine CT, and MRI within a 30-day period were identified. C2–C7 Cobb angles were measured on each modality. Using upright radiographs as the reference standard, the predictive capability of both supine CT and supine MRI in classifying kyphotic versus non-kyphotic alignment was calculated. *Results*: Standing radiographs demonstrated significantly greater lordosis than supine imaging, with mean paired differences of 6.2° versus CT and 5.0° versus MRI (both *p* < 0.001); however, strong correlations were observed (with CT: r = 0.75; with MRI: r = 0.72; both *p* < 0.001). Further, CT-based measurements predicted X-ray Cobb angles with an R^2^ value of 0.57 (estimated X-ray Cobb angle = 8.24 + 0.74 × (CT Cobb angle), β = 0.74, *p* < 0.001). MRI-based measurements yielded an R^2^ of 0.51 (estimated X-ray Cobb angle = 7.59 + 0.71 × (MRI Cobb angle), β = 0.71, *p* < 0.001). At threshold ≥ 0°, CT achieved a 100% NPV for excluding kyphosis on upright radiographs. MRI achieved an NPV of 100% when the Cobb angle was >1.20°. *Conclusions*: Supine CT and MRI systematically underestimate cervical lordosis but demonstrate strong predictive correlation with standing radiographs and reliably exclude true kyphotic alignment, with each achieving near-perfect NPV at defined thresholds. In cases where standing radiographs are unavailable or nondiagnostic, supine imaging modalities such as CT and/or MRI, where the cervical region appears nonkyphotic, can safely rule out cervical kyphosis and inform surgical planning; however, in cases where the cervical region appears kyphotic on CT and/or MRI, standing radiographs remain essential for accurate assessment.

## 1. Introduction

Assessment of cervical lordosis is crucial for surgical planning, predicting postoperative outcomes, and evaluating clinical symptoms in patients with degenerative spinal disorders, particularly when considering laminoplasty or anterior cervical discectomy and fusion (ACDF) [1,2,3]. The most commonly used radiographic method involves measuring the angle formed by two lines drawn parallel to the inferior endplates of the C2 and C7 vertebral bodies. Standing lateral cervical radiographs are widely accepted as the gold standard for this measurement. However, in daily clinical practice, cervical lordosis is often evaluated using imaging modalities other than standing lateral radiographs. Magnetic resonance imaging (MRI) and, less commonly, computed tomography (CT)—both typically obtained in the supine position—are frequently used alternatives for assessing cervical alignment.

In our opinion, there are two main reasons for the frequent use of MRI in assessing cervical lordosis, despite the recognized role of standing lateral radiographs as the gold standard. First, in parallel with the widespread trend toward minimally invasive approaches across all surgical disciplines, there is a growing preference for MRI over direct radiography or CT in radiological evaluations. This shift is largely due to MRI’s advantage of eliminating radiation exposure and its associated risks. Second, and equally important, is the technical limitation commonly encountered in standing lateral radiographs: the C7 vertebra is often difficult to visualize due to shoulder superimposition. Consequently, after obtaining an MRI—which is routinely performed as part of nearly all decision-making processes related to cervical spine pathologies—additional radiograph or CT imaging that involves radiation exposure is often omitted unless necessary. However, there is currently no consensus on how accurately cervical lordosis measured on MRI reflects the measurements obtained from standing plain radiographs [4,5,6,7]. Several studies have reported that factors such as patient positioning, absence of weight bearing, and the use of head immobilization devices during MRI can significantly influence the assessment of cervical lordosis [8,9].

The primary objective of this study is to evaluate whether cervical lordosis angle measurements obtained on cervical MRI and/or CT in the supine position reliably reflect cervical lordosis angle measurements on upright lateral radiographs. The secondary objective is to determine whether MRI and CT scans obtained in the supine position can accurately predict the true cervical alignment (kyphotic or non-kyphotic) seen on radiographs obtained in the upright position.

## 2. Materials and Methods

This retrospective study was approved by the Ethics Committee of Izmir Katip Çelebi University (Date: 17 October 2024, Decision Number: 0127). No identifiable patient information was included; thus, informed consent was not required. Cervical radiological examinations obtained between December 2023 and March 2025 from patients who either presented directly to the Izmir Katip Çelebi University, Ataturk Research and Training Hospital, Department of Neurosurgery, or were referred from other centers with pre-existing imaging, were retrospectively reviewed for this study. In our hospital, imaging can be performed on any of the available scanners (two CT and three MRI machines). The choice of which device to use for each patient was made based on routine clinical practice, without any rules or standardization from the investigators. Radiological examinations of individuals who underwent all three imaging examinations—CT, MRI, and standing lateral plain radiography—for the cervical region were included in the study. A maximum time interval of 30 days between these imaging studies was allowed for inclusion.

### 2.1. Inclusion Criteria

a. Availability of cervical plain radiography, CT, and MRI data for the same individual, all performed within a 30-day period.
b. Age ≥ 18 years.

### 2.2. Exclusion Criteria

a. Cases where the C7 vertebra was not clearly visible on direct radiography due to the shadow cast by the shoulders.
b. All postoperative radiological examinations were excluded from the study.

### 2.3. Cervical Lordosis Angle Measurement

**Figure 1:**
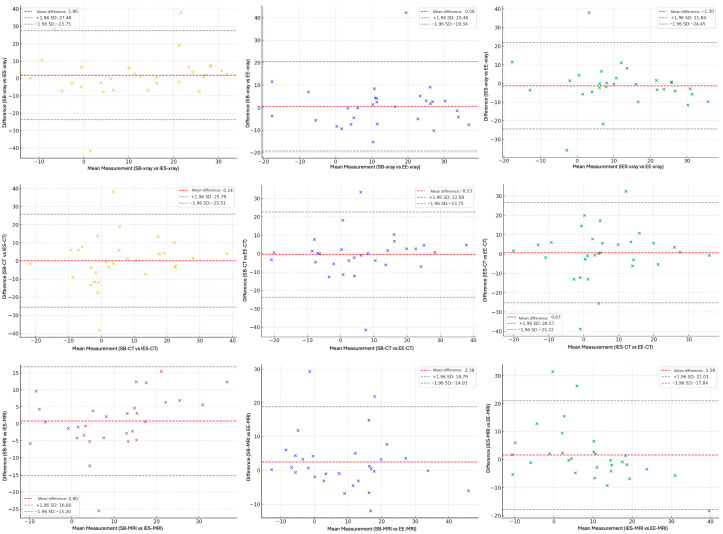
Bland–Altman plots assessing inter-observer agreement across imaging modalities.

All measurements were performed using either the hospital’s PACS system (Probel Inc., Izmir, Türkiye) or the national centralized medical imaging database (e-Nabız, Turkey), depending on the origin of the images. Cobb angles were measured by drawing one line parallel to the inferior endplate of the C2 vertebra and another line parallel to the inferior endplate of the C7 vertebra. The angle formed by the intersection of these two lines was defined as the cervical lordosis angle (Figure 1). Patients in whom the C7 vertebra could not be visualized on standing lateral radiographs, making Cobb angle measurement impossible, were excluded. Based on the C2–C7 Cobb angle, cervical alignment was categorized as kyphotic (<0°) or non-kyphotic (≥0°).

### 2.4. Observer Agreement

First, 30 cases were randomly selected from the entire cohort to assess interobserver reliability. Three neurosurgeons (IES, SB, EE) independently evaluated the cervical lordosis angle in each case using lateral cervical radiographs, CT, and MRI images. Intraclass correlation coefficients (ICCs) were calculated for the Cobb angle measurements across the three imaging modalities. After confirming adequate interobserver agreement, the remaining radiological examinations were divided among the three neurosurgeons for further measurements. To assess intraobserver reliability, the same 30 cases were re-evaluated by each neurosurgeon four weeks after the initial assessment under blinded conditions. ICC values were calculated for each modality to determine intraobserver agreement. The ICC values were interpreted as follows: ≥0.90, excellent; 0.75–0.89, good; 0.50–0.74, moderate; and <0.50, poor. Furthermore, Bland–Altman analysis was conducted to complement agreement assessments between modalities. Mean differences and 95% limits of agreement were calculated and are illustrated using Bland–Altman plots.

### 2.5. Independent Variables

a. Cervical lordosis angle measured on cervical CT (supine position)
b. Cervical lordosis angle measured on cervical MRI (supine position)

### 2.6. Dependent Variables

a. Cervical lordosis angle measured on standing lateral cervical radiographs (reference Cobb angle)

### 2.7. Statistical Analysis

Demographic and quantitative data analyses were performed using IBM SPSS version 27. Statistical significance was defined as *p* < 0.05. Descriptive statistics were reported as means ± standard deviations for Cobb angles measured using the three imaging methods. The normality of continuous variables was assessed using the Shapiro–Wilk test prior to parametric testing. Comparisons of cervical lordosis angles obtained from each imaging modality (plain radiography, CT, and MRI) were conducted using paired *t*-tests as follows:

1. Standing lateral cervical radiographs vs. supine CT
2. Standing lateral cervical radiographs vs. supine MRI

Correlations between cervical lordosis angles measured using plain radiography and those obtained using CT and MRI were assessed using Pearson’s correlation analysis.

Linear regression analysis was performed to evaluate the predictive capability of supine CT and MRI measurements for estimating the standing plain radiography Cobb angle. Regression equations and the goodness-of-fit (R^2^) values were determined to demonstrate the degree of accuracy of these predictions. Regression coefficients (β), standard errors, 95% confidence intervals, and *p*-values were reported.

In addition, the diagnostic accuracy of CT and MRI in classifying cervical alignment (kyphotic vs. non-kyphotic) was evaluated using sensitivity, specificity, positive predictive value (PPV), and negative predictive value (NPV), with standing lateral cervical radiographs considered as the reference standard.

## 3. Results

### 3.1. Observer Reliability

Interobserver reliability was good for standing lateral cervical radiographs and MRI and moderate for CT, while intraobserver reliability remained similarly good/excellent after a four-week interval; ICC values for both assessments are presented in Table 1, and Bland–Altman plots illustrating interobserver agreement are shown in Figure 1.

### 3.2. Study Population and Cervical Parameters

A total of 129 individuals who had undergone all three imaging modalities were retrospectively reviewed. In 11 of these patients, the C7 vertebra could not be visualized on standing lateral radiographs; therefore, the Cobb angle could not be measured. Ten individuals were excluded because only postoperative imaging was available in some modalities, and complete preoperative imaging in all three modalities (X-ray, CT, and MRI) could not be obtained. The final analysis included 108 patients who met the inclusion criteria. Of these, 45 were male (41.7%) and 63 were female (58.3%), with a mean age of 51.3 years (range 18–87 years).

The mean cervical lordosis angle (Cobb angle) measured on standing plain radiographs was 13.9 ± 13.0 degrees (min: −15.8; max: 40.6). The mean cervical lordosis angles measured on supine CT and MRI were 7.7 ± 13.3 (min: −22.5; max: 40.6) and 9.0 ± 13.2 (min: −17.7; max: 48.8), respectively. All three measurements were found to follow a normal distribution according to the Shapiro–Wilk test (X-ray: *p* = 0.460, CT: *p* = 0.272, MRI: *p* = 0.403).

Based on X-ray measurements, 91 patients (84.3%) were classified as non-kyphotic (Cobb angle ≥ 0°), while 17 patients (15.7%) were classified as kyphotic (Cobb angle < 0°). Based on CT measurements, 79 patients (73.1%) were classified as non-kyphotic (Cobb angle ≥ 0°), while 29 patients (26.9%) were classified as kyphotic (Cobb angle < 0°). Similarly, based on MRI measurements, 79 patients (73.1%) were classified as non-kyphotic, while 29 patients (26.9%) were classified as kyphotic.

Overall, standing plain radiographs revealed greater lordosis compared with supine imaging, with a mean difference of 6.21° from CT and 4.95° from MRI (both *p* < 0.001). The distribution of Cobb angles across cervical sagittal alignment groups is presented in Table 2.

### 3.3. Comparisons of Parameters

Significant differences in cervical lordosis measurements were observed across imaging modalities (Table 2). The mean paired differences between radiographs and CT (6.21°) and between radiographs and MRI (4.95°) were both highly significant (*p* < 0.001 for both comparisons). When analyses were stratified by alignment group, similar patterns emerged in the non-kyphotic subgroup. In the kyphotic subgroup, the paired difference between radiographs and CT remained significant (*p* = 0.001), whereas the mean difference between radiographs and MRI (0.73°) did not reach significance (*p* = 0.675).

### 3.4. Correlations of Parameters

Pearson’s correlation analysis revealed a strong and statistically significant correlation between cervical lordosis measurements from standing plain radiographs and supine CT (r = 0.75, *p* < 0.001) and MRI (r = 0.72, *p* < 0.001). To further evaluate the predictive capability of supine imaging modalities, linear regression analyses were performed. CT-based measurements predicted X-ray Cobb angles with an R^2^ value of 0.57. The corresponding regression equation was as follows:

- Estimated X-ray Cobb angle = 8.24 + 0.74 × CT Cobb angle, with a β coefficient of 0.74 (95% CI: 0.61–0.86, SE = 0.062, *p* < 0.001)

For MRI, the regression model yielded an R^2^ of 0.51 and the following equation:

- Estimated X-ray Cobb angle = 7.59 + 0.71 × MRI Cobb angle, with a β coefficient of 0.71 (95% CI: 0.57–0.84, SE = 0.067, *p* < 0.001)

### 3.5. Diagnostic Accuracy

**Figure 2:**
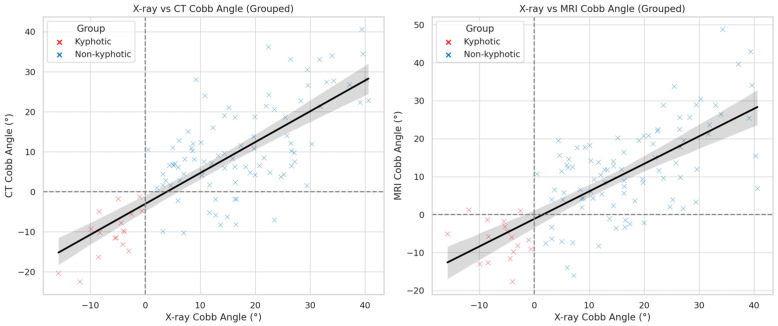
Correlations of CT and MRI Cobb angles with X-ray measurements, grouped by cervical alignment. Scatter plots with regression lines demonstrating the correlation between standing X-ray Cobb angles and those measured using CT (left) and MRI (right). Patients were categorized into kyphotic (Cobb angle < 0°, red) and non-kyphotic (Cobb angle ≥ 0°, blue) groups based on X-ray measurements. Dashed lines indicate the 0° threshold used to define kyphosis. Solid black lines represent the overall linear regression fit. The shaded gray area surrounding each regression line denotes the 95% confidence interval. The graphs illustrate the relationship between supine imaging modalities and upright reference values across both alignment groups.

To assess the ability of CT and MRI to correctly classify cervical alignment (kyphotic vs. non-kyphotic), diagnostic accuracy metrics were calculated using standing lateral radiographs as the reference standard. At a threshold of ≥0°, CT demonstrated a sensitivity of 100%, specificity of 86.8%, positive predictive value (PPV) of 58.6%, and negative predictive value (NPV) of 100% (Table 3). Importantly, no patient classified as non-kyphotic on CT (Cobb angle ≥ 0°) was found to be kyphotic on standing radiography, indicating that a normal CT virtually rules out the presence of kyphosis (Figure 2).

**Figure 3:**
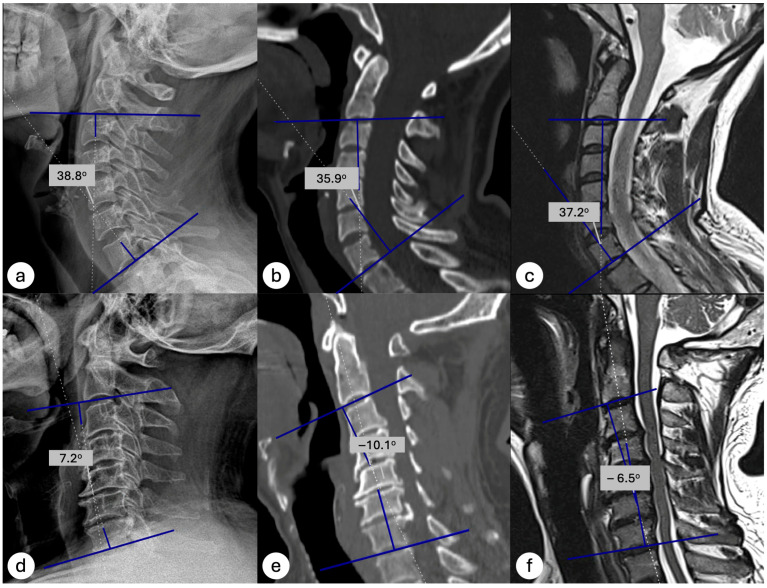
Demonstrative cases of cervical lordosis measurement across imaging modalities. Top row (Case 1): Cobb angle measurements on standing lateral radiograph (**a**), supine CT (**b**), and supine MRI (**c**) demonstrate lordotic alignments of 38.8°, 35.9°, and 37.2°, respectively, classifying the case as non-kyphotic in each modality. Bottom row (Case 2): The patient’s supine CT (−10.1°) (**d**) reveals a non-kyphotic, lordotic curvature, illustrating how cervical lordosis can appear diminished or reversed on supine imaging but re-emerge under weight-bearing conditions (**e**) and supine MRI (−6.5°) (**f**) both show kyphotic alignment, whereas the standing lateral radiograph (7.2°).

MRI showed comparable diagnostic values, with a sensitivity of 88%, specificity of 85%, PPV of 52%, and NPV of 98% when the standard threshold (0°) was used. Notably, when applying a threshold of >1.20°, MRI achieved a 100% NPV, meaning that no patient with a Cobb angle greater than 1.20° on MRI was found to have kyphosis on standing radiographs (Table 4). Demonstrative case examples are shown in Figure 3. These results suggest that both modalities are highly reliable in excluding kyphotic alignment, but confirmation with upright radiographs is required when kyphosis is suggested on supine imaging.

## 4. Discussion

In this retrospective analysis comparing standing lateral radiographs with supine CT and MRI for C2–C7 Cobb angle measurement, the regression analysis provides a quantitative framework for translating supine imaging measurements into clinically meaningful estimates of upright cervical alignment. Both CT and MRI demonstrated strong correlations with standing radiographs (r = 0.75 and r = 0.72, respectively; *p* < 0.001), closely mirroring Goh et al.’s finding of an r = 0.76 correlation between standing radiographs and MRI (*p* = 0.46) [10]. Regression modeling further confirmed their predictive value (CT, R^2^ = 0.57; MRI, R^2^ = 0.51). The derived regression equations offer clinicians an approximate method for estimating true standing alignment from supine studies. For example, if cervical lordosis is measured as +10° on supine MRI, the corresponding upright Cobb angle can be predicted as approximately +14.7°, with a 95% confidence interval suggesting that the true value is most likely between +8° and +19°. Such predictive ranges highlight that although CT and MRI cannot fully substitute for standing radiographs, they provide clinically meaningful estimations of upright alignment, especially when radiographs are unavailable, incomplete, or nondiagnostic.

The second key finding of this study is that both supine modalities reliably exclude, but cannot definitively identify, cervical kyphosis. CT-based measurements ≥ 0° demonstrated a negative predictive value (NPV) of 100% for kyphosis on upright radiographs, while MRI Cobb angles > 1.20° likewise achieved an NPV of 100%. In other words, whenever supine CT (≥0°) or MRI (>1.20°) indicated non-kyphotic alignment, no patient was subsequently found to be kyphotic on standing radiographs. By contrast, the positive predictive values were modest—58.6% for CT and 52% for MRI—highlighting the limited ability of supine imaging to confirm true kyphosis in cases that appear kyphotic when unloaded. These findings emphasize that, while supine CT and MRI cannot replace standing radiographs for the definitive diagnosis of kyphosis, they provide a reliable means to safely rule out kyphotic alignment when upright imaging is unavailable, incomplete, or infeasible.

The finding that supine CT and MRI measurements reliably exclude cervical kyphosis aligns with and extends prior studies examining the influence of patient position on cervical alignment. Boudreau et al. reported in a cohort of myelopathy patients that cervical lordosis angles on supine MRI were, on average, a few degrees smaller than on standing radiographs, and notably, 15% of patients who appeared to have no lordosis on supine MRI did show a lordotic curvature when imaged upright [6]. This can be explained by increased gravitational loading and active paraspinal muscle engagement in the standing posture. In our cohort, the mean paired differences in Cobb angle—4.95° for CT and 6.21° for MRI—were lower than those on standing lateral radiographs, indicating that supine imaging reduces cervical lordosis. These observations are consistent with those of several studies that reported that key alignment measures, such as C2–7 Cobb angle, were significantly lower on supine MRI compared with standing radiographs [4,8,11].

In our cohort’s non-kyphotic subgroup, standing radiographs showed significantly greater cervical lordosis than supine MRI (mean difference 5.74°; *p* < 0.001), whereas in the kyphotic subgroup, the difference was non-significant (0.73°; *p* = 0.675). In contrast, supine CT showed significantly lower cervical lordosis than standing radiographs in both the kyphotic and non-kyphotic subgroups. Similarly, Oshina et al. found no significant difference in the C2–7 Cobb angle between upright X-ray and supine MRI in patients with cervical kyphosis. In other words, true kyphotic alignment was similarly evident on supine MRI for these patients. By contrast, in lordotic patients, the standing radiograph showed ~4.5° greater lordosis than supine MR [7]. The authors have proposed two potential mechanisms for this. First, patients with true cervical kyphosis often have advanced disk degeneration and segmental collapse, which limit motion and lead to more consistent alignment readings; in contrast, those with preserved lordosis typically have fewer degenerative changes and greater segmental flexibility. Second, in lordotic spines—particularly when the thoracic region is flexible—the transition to supine can flatten the thoracic curve, causing the occiput to contact the scanner bed and passively force the cervical spine toward a more kyphotic posture during imaging. These pathophysiological explanations are also supported by prior studies reporting that advanced degeneration stabilizes kyphotic spines, whereas the greater flexibility of lordotic spines makes their alignment more posture-dependent [12,13].

Clinically, our results offer a pragmatic approach when standing lateral radiographs are unavailable or nondiagnostic. This often occurs in trauma or preoperative settings, where a patient may undergo CT and MRI for injury or pathology evaluation before any standing films are obtained. Standard lateral cervical radiographs also often fail to visualize the C7 vertebra‘s lower endplate due to shoulder obstruction. Indeed, one study noted that only approximately 21% of cervical radiographs had a clear view of the T1 vertebral body [14]. In these situations, surgeons often rely solely on supine CT or MRI for preoperative planning. By applying a simple Cobb angle threshold (≥0° on CT or >1.20° on MRI), clinicians can confidently rule out true kyphosis and proceed with surgical strategies without undue concern for occult malalignment. However, cases appearing kyphotic on supine imaging warrant confirmatory upright radiographs, given the lower positive predictive value.

Karabag et al. proposed that MRI images obtained using a 5 cm high headrest best represent standing direct radiographs in terms of lordosis angle measurement [9]. However, in daily practice, physicians working in the periphery or in private practice usually do not know how the MRI images were taken. Moreover, they often have no opportunity to guide the technique used for the acquisition of these images. Therefore, we need to be able to form an opinion about how well MRI images obtained from various centers can represent standing direct radiographs, regardless of how they are taken. It is logical to think that this representation rate in such a situation is lower than when the imaging techniques are under our own control; however, this is the exact situation encountered in real life. In contrast to prior studies relying on a single scanner or institution [8], our cohort comprised images acquired on a variety of platforms, both from patients admitted directly to our clinic and those referred from multiple centers. Although this approach introduces inter-scanner variability and could be seen as a limitation, it accurately reflects real-world imaging practices and therefore strengthens the external validity and generalizability of our findings.

This study had several limitations. Although we restricted the time interval between the imaging modalities to within one month, the possibility of day-to-day variation in patient posture and comfort remains. A patient’s pain level or muscle spasm on the day of imaging could have subtly influenced their posture; for instance, the lordosis measurement could have been affected if a patient was stiff during the X-ray but more relaxed during the MRI a week later. Second, this study did not report detailed clinical information, such as the patients’ weight/height and chief complaints, which may have affected the alignment. Third, our analysis focused on neutral alignment (the standard upright lateral view and the supine neutral position in CT/MRI). We did not evaluate dynamic alignment changes on flexion–extension radiographs.

## 5. Conclusions

Although supine CT and MRI measurements underestimate cervical lordosis compared with standing radiographs, they show a strong correlation from which the estimated standing X-ray Cobb angle can be calculated; furthermore, they can reliably exclude true kyphotic alignment. A CT Cobb angle ≥ 0° and MRI > 1.20° demonstrated 100% NPV for true kyphosis, supporting their use when upright imaging is unavailable or nondiagnostic. These supine modalities offer a practical tool for excluding kyphosis and informing surgical planning; however, in cases with suspected kyphotic alignment, standing radiographs remain essential for accurate assessment.

## Figures and Tables

**Table 1:** Interobserver and intraobserver reliability.

Imaging Modality	Interobserver Reliability *	Intraobserver Reliability *		
		SB	IES	EE
**Radiograph**	0.81	0.83	0.72	0.91
**CT**	0.72	0.78	0.78	0.77
**MRI**	0.88	0.93	0.80	0.83

* ICC values.

**Table 2:** Cervical Cobb angle measurements.

	Radiography (Mean ± SD)	CT (Mean ± SD)	MRI (Mean ± SD)	Paired Difference in the Mean Radiography—MRI (*p*)	Paired Difference in the Mean Radiography—CT (*p*)
**C2–7 angle** **(total)**	13.93 ± 13.02	7.72 ± 13.32	8.98 ± 13.20	4.95 **(<0.001)**	6.21 **(<0.001)**
**C2–7 angle** **non-kyphotic group**	17.65 ± 10.47	11.10 ± 11.47	11.91 ± 12.13	5.74 **(<0.001)**	6.55 **(<0.001)**
**C2–7 angle** **kyphotic group**	−5.99 ± 4.00	−10.35 ± 5.92	−6.73 ± 5.20	0.73 (0.675)	4.36 **(0.001)**

Bold: statistically significant.

**Table 3:** Diagnostic performance of CT at threshold ≥ 0° in classifying cervical alignment (kyphotic vs. non-kyphotic), using standing lateral cervical radiographs as the reference standard.

	X-Ray	Kyphotic	Non-Kyphotic
CT			
Kyphotic < 0°		17 (TP)	11 (FP)
Non-kyphotic ≥ 0°		0 (FN)	80 (TN)

TP: True Positive; FN: False Negative; FP: False Positive; TN: True Negative; Sensitivity = 100%; Specificity = 86.8%; PPV = 58.6%; NPV = 100%.

**Table 4:** Diagnostic performance of MRI in classifying cervical alignment (kyphotic vs. non-kyphotic), using standing lateral cervical radiographs as the reference standard.

	X-Ray	Kyphotic	Non-Kyphotic
MRI			
Kyphotic < 0°		15 (TP)	14 (FP)
Non-kyphotic ≥ 0°		2 (FN)	77 (TN)
MRI > 1.20°			
≤1.20°		17 (TP)	14 (FP)
>1.20°		0 (FN)	77 (TN)

TP: True Positive; FN: False Negative; FP: False Positive; TN: True Negative; Threshold 0°; Sensitivity = 88%; Specificity = 85%; PPV = 52%; NPV = 98%; Threshold 1.2°; Sensitivity = 100%; Specificity = 85%; PPV = 55%; NPV = 100%.

## Data Availability

The data presented in this study are available from the corresponding author upon reasonable request in accordance with ethical and privacy restrictions.

## Abbreviations

The following abbreviations are used in this manuscript:

CT	Computed Tomography
MRI	Magnetic Resonance Imaging
ICC	Intraclass Correlation Coefficient
PPV	Positive Predictive Value
NPV	Negative Predictive Value
TP	True Positive
TN	True Negative
FP	Fase Positive
FN	False Negative
CI	Confidence Interval
SD	Standard Deviation
